# Comparative Long-Term Outcomes of Pulsed and Lesion Radiofrequency of the Greater Occipital Nerve in Chronic Migraine: A 12-Month Cohort Study

**DOI:** 10.3390/medicina61111893

**Published:** 2025-10-22

**Authors:** Ahmet Yilmaz, Cagatay Kucukbingoz

**Affiliations:** Department of Algology, Adana City Training and Research Hospital, Adana 01370, Türkiye; ckbingoz.md@gmail.com

**Keywords:** chronic migraine, radiofrequency ablation, pulsed radiofrequency treatment, greater occipital nerve, pain management, treatment outcome, quality of life, safety

## Abstract

*Background and Objectives:* Chronic migraines are a disabling neurological disorder with limited response to preventive pharmacological treatments. Greater occipital nerve (GON)-targeted radiofrequency (RF) procedures have emerged as promising interventions, yet long-term comparative data between pulsed RF (PRF) and continuous-lesion RF (LesionRF) remain scarce. This study evaluated the 12-month efficacy and safety of PRF versus LesionRF. *Materials and Methods:* A single-center cohort of 211 patients with chronic migraine diagnosed by ICHD-3 criteria (PRF = 107; LesionRF = 104) was analyzed. All patients had a positive diagnostic block and ≥12 months of follow-up. Interventions were performed under ultrasound guidance with standardized protocols (PRF: 42 °C, 4 min, 45 V; LesionRF: 80 °C, 90 s). The primary outcome was a change in monthly migraine days (MMD), while secondary outcomes included responder rates (≥50% MMD reduction), pain intensity (VAS), functional outcomes (HIT-6, MIDAS), quality of life (SF-36, EQ-5D), medication use, retreatment, and complications. *Results*: Both groups improved, but LesionRF showed greater benefit. At 12 months, LesionRF achieved a larger MMD reduction (−4.8 days vs. PRF, *p* < 0.001), higher responder rates (83% vs. 65%, *p* = 0.01), and greater VAS decreases (−1.6, *p* < 0.001). Functional and quality-of-life scores improved more with LesionRF, with MIDAS reductions surpassing MCID and responder rates meeting PASS. Retreatment was less frequent with LesionRF (8% vs. 19%; HR 2.15, *p* = 0.037), and two LesionRF patients (1.9%) developed hematomas that resolved conservatively. *Conclusions*: Compared with PRF, LesionRF provided more sustained and clinically meaningful benefits for chronic migraines. Both approaches appeared to be safe, though confirmation in larger randomized trials is warranted.

## 1. Introduction

Chronic migraines impact roughly 1–2% of the global population, with a significant female predominance, and are among the foremost neurological illnesses contributing to years lived with disability globally [1]. In addition to the personal toll of repeated headaches, chronic migraines incur considerable socioeconomic expenses due to work absenteeism, heightened healthcare usage, and a marked decline in quality of life [2]. Notwithstanding the existence of preventive pharmaceutical interventions, empirical data reveal that less than 40–50% of patients experience lasting positive responses, while dropout rates persist at elevated levels due to unpleasant effects [3]. As a result, interventional methods have garnered heightened interest in refractory instances.

The greater occipital nerve (GON) blocks are the most well researched among these options [4]. While local anesthetic and corticosteroid injections offer temporary relief, their effectiveness often endures for only a few weeks [5]. This limitation has required the adoption of radiofrequency (RF)-based neuromodulation techniques. Pulsed RF (PRF) delivers short bursts of electrical fields at low temperatures, modulating axonal transmission and minimizing tissue damage. Initial evidence suggests that PRF is safe; nonetheless, its analgesic effects are often transient [6]. In contrast, continuous thermal lesion radiofrequency (LesionRF) use high temperatures to generate neurodestructive lesions, potentially offering longer lasting pain relief; however, safety concerns, particularly regarding outcomes, remain unresolved [7]. The LesionRF parameters utilized in this investigation (80 °C for 90 s) were chosen based on previous evidence demonstrating an appropriate equilibrium between efficacy and safety [6,7]. Moreover, existing clinical guidelines emphasize the necessity of enhancing preventative efforts for chronic migraine, highlighting the demand for innovative interventional methods [8].

In addition, the literature indicates that greater occipital nerve (GON) blocks have demonstrated therapeutic benefit in both migraines with aura and without aura when assessed in separate cohorts. Furthermore, small-scale studies have also suggested the analgesic efficacy of GON blocks in patients with tension-type headache, thereby broadening the potential scope of this intervention [9,10,11,12].

The current evidentiary base is insufficient. The majority of published trials are single-center, involve small cohorts (fewer than 50 patients), and report only short-term outcomes (3–6 months) [9]. Direct long-term comparative data on PRF versus LesionRF are nearly nonexistent. Moreover, essential patient-centered outcomes, including responder rates, functional impairment, quality of life, necessity for retreatment, and complication profiles, have frequently been inadequately documented. Clinically significant thresholds, such as the Minimal Clinically Important Difference (MCID) and the Patient Acceptable Symptom State (PASS), have infrequently been utilized, rendering the practical significance of findings ambiguous [13,14].

This study aims to directly compare PRF and LesionRF in patients with chronic migraine, with a follow-up period of 12 months. We assessed efficacy, safety, functional outcomes (HIT-6, MIDAS), quality-of-life metrics (SF-36, EQ-5D), medication utilization, retreatment frequencies, and complication profiles.

Primary Hypothesis: In comparison to PRF, LesionRF is anticipated to yield a more significant and clinically relevant decrease in monthly migraine days (MMD) at the 12-month mark.

Secondary Hypotheses: LesionRF is anticipated to correlate with elevated responder rates, more significant reductions in pain intensity (VAS), prolonged enhancements in functional and quality-of-life metrics, diminished medication consumption, and decreased retreatment frequencies, while both methodologies are expected to exhibit an acceptable safety profile.

## 2. Materials and Methods

### 2.1. Study Design and Clinical Practice

This study was conducted as a single-center retrospective cohort analysis. All interventions were performed at the Pain Clinic of Adana City Training and Research Hospital between 2022 and 2023, in accordance with global guidelines and established institutional regulations. The study’s retrospective design excluded randomization and blinding, constituting a possible source of bias. To alleviate selection bias, only patients with a confirmed diagnostic block were included, procedural preferences were standardized, and all therapies were performed under the oversight of the same seasoned pain specialists.

### 2.2. Ethical Approval

The study was conducted in accordance with the Declaration of Helsinki [15]. Ethical permission was obtained from the Adana City Training and Research Hospital Ethics Committee (protocol number: 58; date: 26 June 2024). The study’s retrospective approach eliminated the requirement for formal informed consent.

### 2.3. Participants

Inclusion criteria: An additional criterion was the absence of prior interventional treatment for migraines (e.g., nerve blocks, botulinum toxin injections, or radiofrequency procedures).

Pharmacological treatment resistance was defined as prior inadequate response or intolerance to at least one standard preventive medication (e.g., amitriptyline, topiramate, valproate, gabapentin, propranolol, flunarizine).

The inclusion criteria comprised patients with a diagnosis of chronic migraine according to the International Classification of Headache Disorders, 3rd edition (ICHD-3), defined as ≥15 headache days per month, of which ≥8 fulfilled migraine criteria. Eligible patients were between 18 and 75 years of age, had undergone a positive diagnostic block prior to intervention, and had a minimum of 12 months of available follow-up data. In addition, only patients who had no prior interventional treatment for migraine (such as nerve blocks, botulinum toxin injections, or radiofrequency procedures) were included. Pharmacological treatment resistance was defined as an inadequate response or intolerance to at least one standard preventive medication, including amitriptyline, topiramate, valproate, gabapentin, propranolol, or flunarizine.

Patients were excluded if they had secondary headache disorders, a history of previous interventional procedures for migraine, severe coagulopathy, active infection, or major neurological deficits. Individuals with documented major psychiatric disorders interfering with outcome assessment (such as severe depression or bipolar disorder) were also excluded. Furthermore, cases with incomplete or insufficient clinical records that prevented reliable data collection were not eligible.

A total of 211 patients were included (PRF = 107; LesionRF = 104).

### 2.4. Interventional Procedures

All treatments were conducted in a specialized interventional pain suite utilizing ultrasound guidance, accompanied by standard patient monitoring and stringent aseptic protocols. After local skin preparation, infiltration of local anesthetic at the puncture site, and sterile draping, the greater occipital nerve (GON) was identified using a 22-gauge RF cannula. RF procedures were performed using a Cosman™ G4 RF Generator (Boston Scientific, Burlington, MA, USA) with 22 G RF cannulas (10 mm active tip). In the PRF group, settings were established at 42 °C for 4 min at 45 V. In the LesionRF group, constant thermal lesioning was administered at 80 °C for 90 s. Interventions were executed unilaterally or bilaterally based on clinical indications, with all procedures carried out by the same team of seasoned pain specialists to maintain uniformity. The chosen parameters were obtained from previous research on chronic headache and occipital nerve treatments [16,17]. All procedures were performed by algology specialists with at least 5 years of interventional pain management experience.

### 2.5. Outcome Measures

The primary outcome of the study was the change in monthly migraine days (MMD) from baseline to 3, 6, 9, and 12 months of follow-up. Secondary outcomes included the responder rate, defined as a ≥50% reduction in MMD compared with baseline, and pain intensity, assessed using the Visual Analog Scale (VAS) [18,19]. Functional outcomes were evaluated with the Headache Impact Test-6 (HIT-6) [20], while migraine-related disability was assessed using the Migraine Disability Assessment (MIDAS) questionnaire [21]. Quality of life was measured with the Short Form-36 Health Survey (SF-36) [22] and the EuroQol-5 Dimensions (EQ-5D) instrument [23]. Additional measures included attack characteristics such as mean attack duration and weekly attack frequency, as well as medication use, defined as the proportion of patients using preventive or acute medications at baseline and at the 12-month visit. Retreatment rates and time to retreatment were recorded, and safety outcomes encompassed both minor complications (e.g., injection-site pain, transient dizziness) and major complications (e.g., hematoma, neurological deficits).

All scales used in this study (VAS, HIT-6, MIDAS, SF-36, EQ-5D) are validated instruments. VAS and the RAND-SF-36 version are generally available for noncommercial academic use without license, whereas HIT-6, MIDAS, and EQ-5D were used under academic, non-commercial research protocols according to the usage policies of their developers.

### 2.6. Follow-Up

Patients were evaluated on day 15, and at months 1, 3, 6, 9, and 12. All follow-ups were conducted through face-to-face visits by an independent pain physician not involved in the interventions. Telephone interviews or patient diaries were not used, which may introduce recall bias and should be considered a study limitation. During follow-up, attrition was 6% (PRF: 92; LesionRF: 93–94). Baseline characteristics of patients lost to follow-up did not differ significantly between groups, suggesting no substantial risk of attrition bias. The number of days on which patients experienced headaches were self-reported during clinical visits. This reliance on patient recall represents a methodological limitation. All clinical scales (VAS, HIT-6, MIDAS, SF-36, EQ-5D) were assessed by two independent, experienced algology specialists to minimize observer bias [24].

### 2.7. Statistical Analysis

Data were analyzed using IBM SPSS Statistics version 28.0 (IBM Corp., Armonk, NY, USA) and R software version 4.2.2 (R Foundation for Statistical Computing, Vienna, Austria). Descriptive statistics were reported as Comparisons between groups were performed using independent-samples *t* tests or Mann–Whitney U tests for continuous variables, and chi-square testing for categorical data. Longitudinal alterations were assessed utilizing linear mixed models incorporating group × time interaction variables. Response rates were evaluated using chi-square testing, and odds ratios with 95% confidence intervals (CIs) were computed. Attack duration and frequency were assessed by independent-samples *t* tests, whereas functional and quality-of-life measures were evaluated based on mean differences, accompanied by corresponding 95% confidence intervals and *p* values. Alterations in medication utilization were analyzed using two-proportion z tests. Retreatment rates were evaluated utilizing Kaplan–Meier survival curves and log-rank testing, with hazard ratios (HRs) and 95% confidence intervals (CIs) presented alongside the median time to retreatment. The incidence of missing data was 6%, predominantly impacting MMD, VAS, and functional scores. Alongside complete-case analysis, multiple imputation using chained equations (MICE) was executed with 20 imputed datasets, and results were aggregated in accordance with Rubin’s guidelines.

No clinically meaningful differences were observed between imputed and complete-case analyses. To enhance transparency, Supplementary Table S1 presents comparisons between complete-case and imputed datasets (MMD, VAS, HIT-6, MIDAS).

No formal a priori power analysis was performed due to the retrospective design. However, post hoc analyses indicated that the available sample size provided >80% power to detect medium-to-large effect sizes (Cohen’s d = 0.5–0.8), supporting the adequacy of the cohort for the primary endpoint. Propensity score methods such as matching or IPTW were not applied due to limited availability of time-varying variables in the retrospective dataset. However, baseline demographic and clinical features were balanced between groups. Post hoc power analysis confirmed that the available sample size (n = 211) provided >80% power to detect medium-to-large effect sizes (Cohen’s d = 0.5–0.8), consistent with recommendations from prior methodological studies [25,26].

### 2.8. Subgroup Analyses

Subgroup analyses were conducted according to sex (male/female) and comorbidities (e.g., anxiety, fibromyalgia, obesity, hypertension). Responder rates and MMD changes were reported separately for these subgroups. These analyses were predefined but considered exploratory and hypothesis-generating. Formal multiple-comparison correction (e.g., Bonferroni adjustment) was not applied due to the limited sample sizes, and findings should therefore be interpreted with caution.

### 2.9. Safety Monitoring

All complications were systematically assessed during the first week and at 1 month; no delayed complications were reported thereafter. Two major complications (hematomas) were observed in the LesionRF group; both resolved completely with conservative management within approximately four weeks, leaving no sequelae. No neurological deficits, sensory loss, or infections were identified. Minor adverse events such as injection-site pain and transient dizziness occurred in approximately 5% of patients. Complications rarely reported in the literature, including transient hypoesthesia or paresthesia, were not observed.

## 3. Results

**Figure 1:**
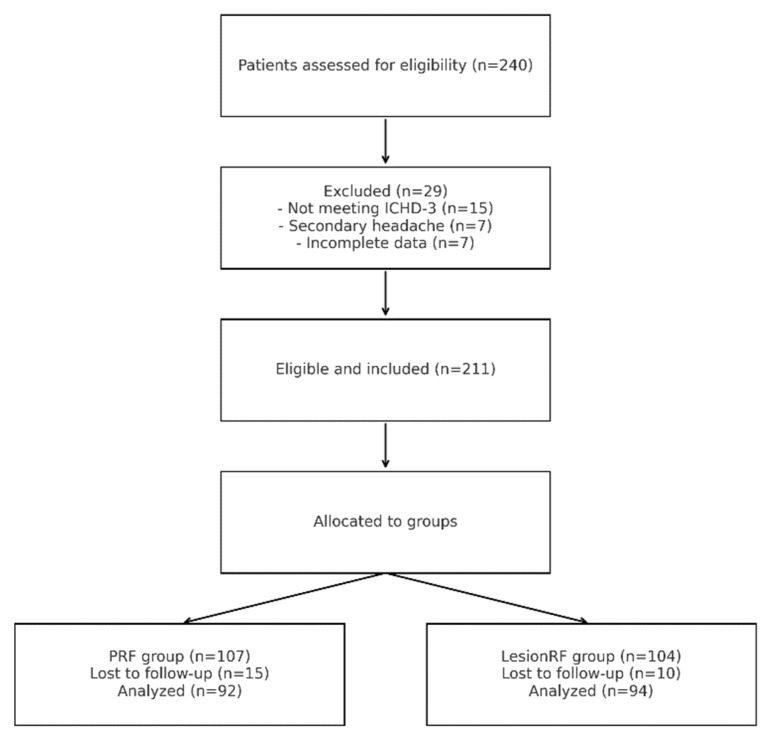
Patient flow diagram. Legend: The figure illustrates the flow of patients throughout the study. Screening, exclusion criteria, non-randomized allocation into PRF and LesionRF groups, and the number of patients analyzed at 12 months are presented. Abbreviations: PRF = pulsed radiofrequency; LesionRF = lesion radiofrequency; ICHD-3 = International Classification of Headache Disorders, 3rd edition.

A total of 211 patients were included (PRF = 107; LesionRF = 104). Baseline characteristics, including age, sex, migraine duration, body mass index (BMI), and comorbidity profiles, were balanced between groups (Table 1). The mean age was 40.6 ± 11.3 years in the PRF group and 39.5 ± 11.0 years in the LesionRF group (*p* = 0.48). Approximately 70% of participants in each group were women (*p* = 0.91). The mean migraine duration was about 10 years, and the mean BMI was 25–26 kg/m^2^. No significant between-group differences were observed in comorbidities (*p* > 0.05). Overall attrition during follow-up was 6%. At 3, 6, 9, and 12 months, the number of patients analyzed was 92 in the PRF group and 94 in the LesionRF group (Figure 1).

**Figure 2:**
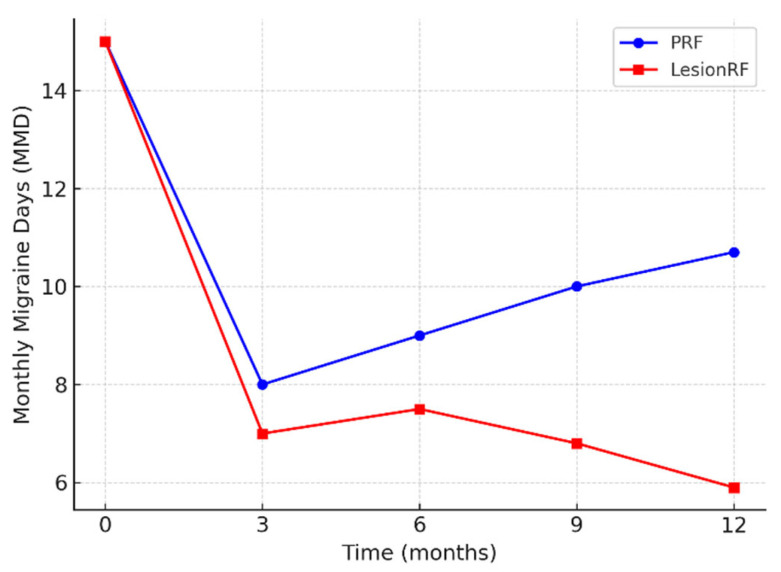
Change in monthly migraine days (MMD). Legend: The figure shows the change in monthly migraine days (MMD) over 12 months in the PRF and LesionRF groups. LesionRF demonstrated a more pronounced reduction at 9 and 12 months compared with PRF. Abbreviations: MMD = monthly migraine days; PRF = pulsed radiofrequency; LesionRF = lesion radiofrequency; CI = confidence interval.

**Figure 3:**
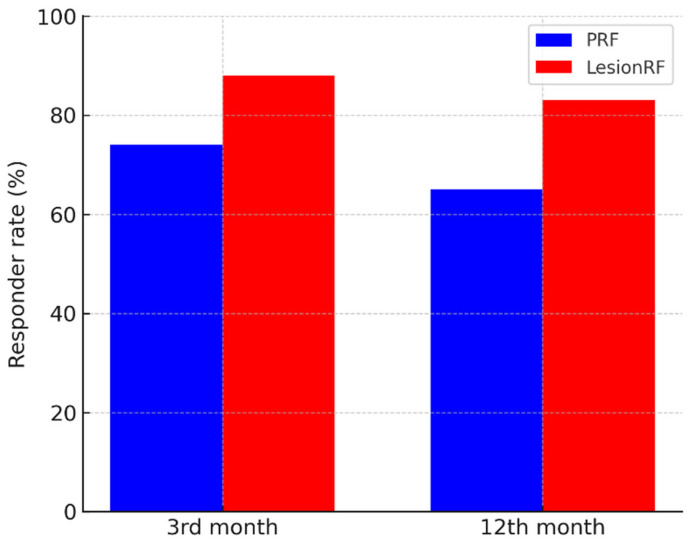
Responder rates (≥50% reduction in MMD). Legend: The figure demonstrates responder rates at months 3 and 12. A greater proportion of patients in the LesionRF group achieved ≥50% reduction in MMD compared with PRF. Abbreviations: MMD = monthly migraine days; PRF = pulsed radiofrequency; LesionRF = lesion radiofrequency.

Monthly migraine days (MMD) decreased significantly in both groups; however, the reduction was more pronounced in the LesionRF group at 9 and 12 months. At 12 months, mean MMD was 10.7 ± 3.1 days in the PRF group and 5.9 ± 2.8 days in the LesionRF group. The between-group difference was −4.8 days (95% CI: −5.9 to −3.7; *p* < 0.001, LMM analysis). The responder analysis (≥50% reduction in MMD) demonstrated a similar advantage for LesionRF: at 3 months, responder rates were 74% (95% CI: 65–82) for PRF and 88% (81–94) for LesionRF (*p* = 0.02); at 12 months, responder rates were 65% (56–74) and 83% (75–89), respectively (*p* = 0.01) (Table 2, Figure 2 and Figure 3).

**Figure 4:**
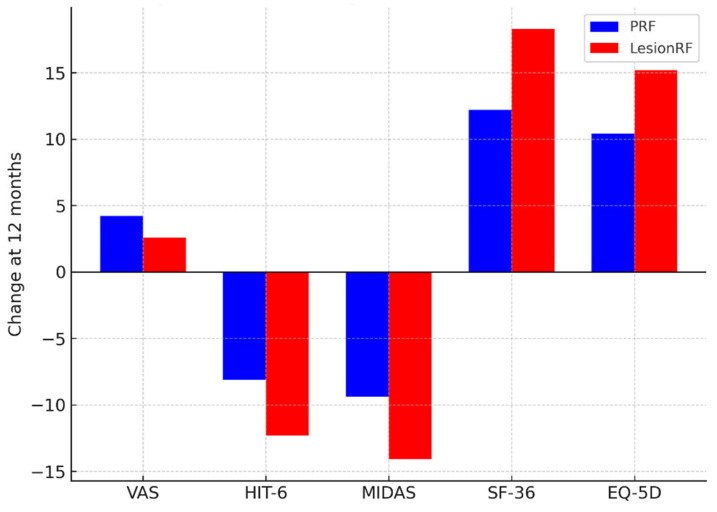
Secondary outcomes at 12 months. Legend: The figure presents changes in pain intensity (VAS), functional disability (HIT-6, MIDAS), and quality-of-life measures (SF-36, EQ-5D) at 12 months. LesionRF yielded greater improvements across all measures compared with PRF. Abbreviations: VAS = Visual Analog Scale; HIT-6 = Headache Impact Test-6; MIDAS = Migraine Disability Assessment; SF-36 = Short Form-36 Health Survey; EQ-5D = EuroQol-5 Dimensions; PRF = pulsed radiofrequency; LesionRF = lesion radiofrequency.

Pain intensity (VAS) scores decreased significantly in both groups, with a greater reduction observed in the LesionRF group. At 12 months, the mean VAS score was 4.2 ± 1.1 in the PRF group and 2.6 ± 0.9 in the LesionRF group (difference −1.6; 95% CI: −2.1 to −1.1; *p* < 0.001). Functional and quality-of-life measures showed a similar pattern. At 12 months, HIT-6 scores decreased by −8.1 in the PRF group versus −12.3 in the LesionRF group (*p* = 0.002); MIDAS scores decreased by −9.4 versus −14.1, respectively (*p* = 0.004). SF-36 scores increased by +12.2 in PRF and +18.3 in LesionRF (*p* = 0.001), and EQ-5D index scores increased by +10.4 versus +15.2 (*p* = 0.003) (Table 3, Figure 4).

Attack characteristics also improved in both groups. Mean attack duration decreased by −5.7 ± 2.5 h in PRF and −8.6 ± 4.4 h in LesionRF (*p* < 0.001). Weekly attack frequency decreased by −0.93 ± 0.72 in PRF and −1.62 ± 0.96 in LesionRF (*p* < 0.001) (Table 4).

At the 12-month follow-up, preventive medication use decreased from 59.8% to 34.6% (–25.2%) in the PRF group and from 54.8% to 27.9% (−26.9%) in the LesionRF group (*p* = 0.29). The use of acute medications declined from 60.7% to 41.1% (−19.6%) in the PRF group and from 64.4% to 39.4% (−25.0%) in the LesionRF group (*p* = 0.80). Although between-group differences were not statistically significant, the overall downward trends were consistent with the clinical improvements observed (Table 5).

**Figure 5:**
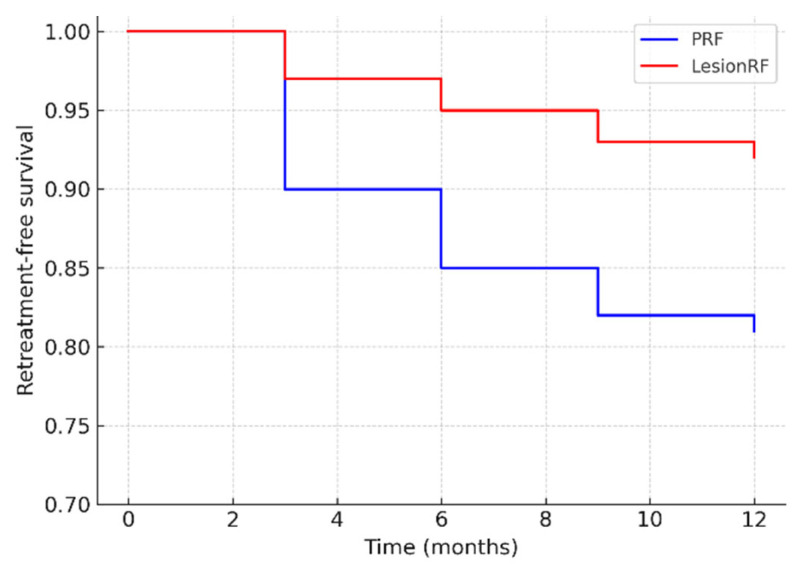
Kaplan–Meier survival analysis for retreatment. Legend: The figure illustrates Kaplan–Meier survival curves for retreatment-free survival over 12 months. Patients in the PRF group had a significantly higher risk of retreatment compared with the LesionRF group. Abbreviations: HR = hazard ratio; CI = confidence interval; PRF = pulsed radiofrequency; LesionRF = lesion radiofrequency.

In the PRF group, the rate was 12–27%, whereas in the LesionRF group, it was 8% (95% CI: 3–14). Kaplan–Meier analysis demonstrated a significantly higher risk of retreatment in the PRF group (log-rank *p* = 0.037; HR 2.15; 95% CI: 1.05–4.41). The hazard ratio indicates that the likelihood of requiring retreatment with PRF was more than twice that observed with LesionRF (Table 6, Figure 5).

**Figure 6:**
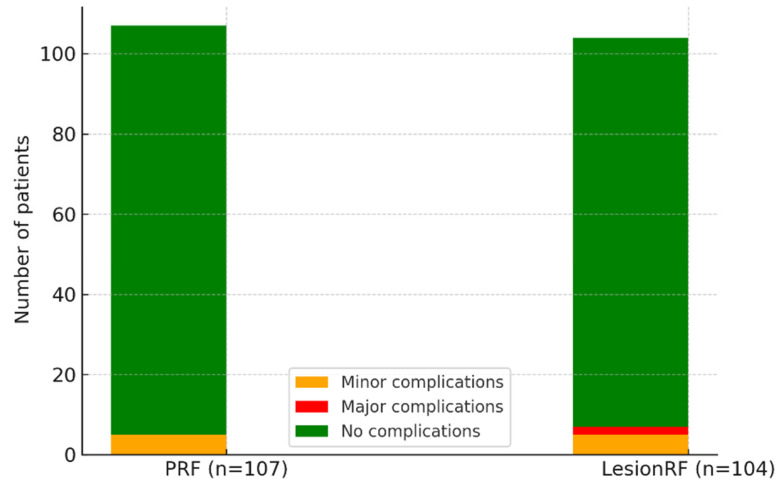
Safety profile. Legend: The figure illustrates the distribution of complications in the PRF and LesionRF groups. Minor complications occurred in approximately 5% of patients in both groups, typically including injection-site pain and transient dizziness. Major complications were observed only in the LesionRF group, where two hematomas (n = 2, 1.9%) developed. Both cases resolved completely with conservative management within four weeks, without sequelae. Abbreviations: PRF = pulsed radiofrequency; LesionRF = lesion radiofrequency.

Adverse events occurred in approximately 5% of patients in each cohort, most commonly presenting as discomfort at the injection site or transient disorientation. Major complications were observed only in the LesionRF group; hematomas developed in two patients (1.9%), which completely resolved within four weeks with conservative management, leaving no sequelae (Table 7, Figure 6).

In the PRF group, response rates were 70% in males and 62% in females (*p* = 0.19). However, efficacy was relatively reduced in patients with anxiety (75%) and fibromyalgia (70%), conditions generally considered treatment-resistant. In the PRF group, response rates in these subgroups were 60% and 55%, respectively (*p* = 0.04 and *p* = 0.05) (Table 8).

## 4. Discussion

Interventional techniques are becoming increasingly significant in the management of chronic migraine, especially in patients resistant to medication. This study directly compared PRF and LesionRF applied to the greater occipital nerve (GON), bridging a significant gap in the literature. The results indicate that LesionRF offers both immediate and prolonged advantages for up to 12 months, exhibiting more significant reductions in headache burden, enhancements in functional outcomes, and reductions in medication usage relative to PRF. Our cohort specifically included patients with chronic migraines refractory to standard pharmacological prophylaxis, including tricyclic antidepressants (e.g., amitriptyline), antiepileptics (e.g., topiramate, valproate, gabapentin), beta-blockers (e.g., propranolol, metoprolol), and calcium-channel blockers (e.g., flunarizine). Interventional approaches were therefore considered only after inadequate response to these established therapies [27].

Monthly migraine days (MMD) and responder rates are essential measures for evaluating interventional efficacy. In our study, LesionRF demonstrated a five-day reduction in MMD and an 83% responder rate at 12 months, in contrast to the 65% responder rate in the PRF group. The results correspond with the randomized controlled experiment conducted by Saraçoğlu et al. (n = 32), which showed more significant MMD reductions when PRF was administered in conjunction with GON block compared to the block alone [24]. Ertilav et al. (n = 67) similarly reported the greater efficacy of PRF compared to repeated blocks, with responder rates surpassing 70% [25]. Our findings augment this evidence foundation by demonstrating that such advantages can endure for up to one year.

Pain intensity (VAS) and functional scores (HIT-6, MIDAS) demonstrated more significant improvements with LesionRF. In the systematic study by Jain et al. (n = 37), PRF considerably lowered VAS scores, although the effects generally persisted for just 4–6 months [26]. Batistaki et al. (n = 57) similarly documented a decrease in VAS from 8.2 to 4.5 at the six-month mark [27]. The decreases noted at 12 months surpassed the standards for clinical significance. Moreover, MIDAS enhancements exceeded MCID thresholds, and responder rates aligned with PASS standards. The findings are consistent with smaller studies, such as Taha et al., which reported reductions in MIDAS scores following PRF [28], and Gürsoy et al. (n = 66), which documented improvements in both MIDAS and depression scores [29]. Overall, the efficacy of PRF has been supported by these small case series; however, the evidence level is low and the generalizability of the results remains limited.

The effect on quality-of-life metrics (SF-36, EQ-5D) was significant. In our sample, LesionRF shown superior enhancements relative to PRF. Kwak et al. (n = 2) previously found substantial enhancements in SF-12 following PRF [30]. The study by Karaduman et al. (n = 39) demonstrated that both unilateral and bilateral pulsed radiofrequency treatments of the greater occipital nerve effectively decreased pain severity and reduced the number of attacks and their length and decreased the need for pain medication [31]. Our study provides distinctive long-term evidence to the literature by showcasing continuous improvements in quality of life over a 12-month period.

Enhancements in attack characteristics were observed in both groups. We noted more pronounced reductions in both attack duration and frequency with LesionRF, corroborating previous findings by Oliveira et al. (n = 24), who documented diminished attack duration and decreased analgesic consumption after ultrasound-guided GON block at three months [32], and Babaoğlu et al. (n = 60), who observed significant declines in attack frequency post-PRF [33]. Although these findings align with the overall efficacy signal, we did not intend to ascertain mechanistic or qualitative alterations in attacks beyond the predetermined objectives in our study; thus, causal inferences concerning attack “quality” should be approached with caution.

Medication utilization diminished in both cohorts, with more significant absolute decreases observed in the LesionRF group. Karaduman et al. (n = 51) observed substantial decreases in analgesic consumption following PRF [34], Turan et al. reported comparable long-term pain relief following ultrasound-guided continuous radiofrequency ablation of the proximal greater occipital nerve [35]. Our findings build upon existing data, indicating that reductions in medication can be maintained for a minimum of 12 months. Differences across groups did not achieve statistical significance (*p* > 0.05); hence, these tendencies should be regarded as supporting rather than confirmatory.

Retreatment rates are essential metrics of long-term viability. In our investigation, retreatment was necessary for 19% of patients receiving PRF compared to 8% of those treated with LesionRF, indicating a significantly elevated retreatment risk for PRF (HR 2.15; 95% CI 1.05–4.41). De Oliveira et al. (n = 608) indicated that both distal and proximal PRF procedures were successful, although the benefits plateaued after three months [32]. Karaduman et al. (n = 39) similarly observed that PRF offered prolonged alleviation compared to blocks [31]. Collectively, these data indicate that LesionRF may diminish the necessity for recurrent treatments over a 12-month period.

The durability of treatment is also an important determinant of cost-effectiveness. In our cohort, the lower retreatment rate in the LesionRF group suggests a potential advantage in terms of healthcare resource utilization. Interventions associated with fewer retreatments, shorter recovery periods, and reduced medication dependence are generally more sustainable and may lower overall healthcare expenditures [36,37]. Desai et al. demonstrated that cooled radiofrequency ablation for chronic knee pain was cost-effective compared with intra-articular hyaluronan injections, providing a real-world model for interventional pain procedures [38]. Similarly, a recent randomized trial analysis reported that radiofrequency denervation for chronic low back pain was both effective and cost-effective compared with placebo [38]. Hambraeus and colleagues reported that radiofrequency neurotomy for chronic spinal pain, despite its relatively high cost per QALY, provides important benchmarks for evaluating interventional pain treatments [39]. Moreover, Abd-Elsayed et al. demonstrated that structured pain management services can be more cost-effective than surgical approaches, with potential savings exceeding USD 8000 per patient in chronic pain populations [40]. Although our study did not perform a formal economic analysis, the lower retreatment rates and reduced medication use observed in the LesionRF group support its potential for improved cost-effectiveness compared with PRF.

The safety profile was generally satisfactory, with minor problems occurring in approximately 5% of both groups. Two hematomas developed in the LesionRF group and completely cleared with conservative management. Despite the absence of neurological sequelae—probably facilitated by image-guided targeting—the trial lacked sufficient power to identify infrequent adverse events, and the exclusive occurrence of hematoma in the LesionRF group necessitates careful interpretation. More extensive multicenter investigations are required to yield more conclusive safety evidence [39,40,41].

Subgroup analyses yield insights regarding clinical heterogeneity. LesionRF demonstrated high response rates in both genders (80–84%), consistent with recent findings from Karaduman et al. (2025) and De Oliveira et al. (2024), which reported comparable analgesic outcomes irrespective of sex [31,32]. PRF, as demonstrated in a prospective series by Pulsed radiofrequency therapy has been reported to provide not only reductions in pain intensity and attack frequency but also improvements in depression and sleep disturbance scores in migraine patients [27]. In our investigation, efficacy was comparatively diminished in patients with anxiety or fibromyalgia, possibly indicating central sensitization and psychosocial stress in these subgroups; due to limited statistical power and absence of multiplicity adjustment, these subgroup results should be regarded as exploratory.

## 5. Limitations

This study possesses several limitations that must be acknowledged when interpreting the results. First, the retrospective single-center cohort design lacked randomization and blinding, raising the possibility of selection, performance, and detection biases. Including only patients with a positive diagnostic block may also have biased the results towards responders and overstated effectiveness compared with an unselected chronic migraine population. Although baseline characteristics were well balanced, residual confounding cannot be excluded, particularly as propensity score methodologies (e.g., matching, weighting) and adjustments for time-varying confounders were not employed.

Second, outcome ascertainment relied on patient recall during in-person consultations, without the use of standardized headache diaries or electronic patient-reported instruments. This may have introduced recall bias and measurement inaccuracies, especially regarding monthly migraine days and attack characteristics. Similarly, preventive and acute medication use was recorded at baseline and at month 12 but not standardized or modeled as time-varying variables. Changes in medication may have influenced the observed outcomes.

Third, although missing data were limited (≈6%) and addressed using multiple imputation (MICE), the assumption that data were missing at random (MAR) may not have held in all cases. Sensitivity analyses beyond complete-case and imputed datasets were not conducted. Subgroup analyses by sex and comorbidity were predefined but exploratory and underpowered, and no multiple-comparison corrections were applied; hence, subgroup findings should be interpreted cautiously and regarded as hypothesis-generating.

Fourth, the safety analysis was limited by sample size. Although complications were rare, the two hematomas observed in the LesionRF group, despite resolving without sequelae, do not establish comparative safety. Adverse events were systematically monitored in the first month, but delayed complications may have been missed.

The generalizability of our findings is limited. All operations were performed under ultrasound direction by skilled pain specialists utilizing predefined parameter settings (PRF: 42 °C/4 min/45 V; LesionRF: 80 °C/90 s). These findings may not apply to fluoroscopy-guided procedures, various cannula designs, lesion dimensions, or parameter regimes. The difference between unilateral and bilateral application may have also affected outcomes. Furthermore, the exclusion of patients with substantial neurological or psychiatric comorbidities improves internal validity but restricts application to actual chronic migraine populations, which frequently present with multiple comorbidities and are managed in specialized referral centers.

Ultimately, although the diminished retreatment rates in the LesionRF group indicate sustainability and possible cost-effectiveness benefits, no formal economic assessment was conducted, rendering any cost conclusions hypothetical. Furthermore, the study lacked preregistration, and while MCID and PASS thresholds were utilized to improve interpretability, these criteria may differ among instruments and groups, hence constraining generalizability across settings. Future prospective, multicenter randomized studies with extended follow-up and integrated health-economic evaluations are necessary to validate and expand these findings.

## 6. Conclusions

This single-center retrospective cohort study revealed that LesionRF had greater sustained effectiveness than PRF in patients with chronic migraines throughout a 12-month follow-up period. Reductions in monthly migraine days, elevated responder rates, and enhanced improvements in pain intensity, functional outcomes, and quality-of-life scores were more significant in the LesionRF group. Reductions in drug utilization and diminished retreatment rates indicate that LesionRF may provide benefits for long-term sustainability.

Both procedures were predominantly safe; however, two hematomas were reported in the LesionRF group, which fully healed with conservative treatment. Due to the study’s sample size, unusual problems could not be thoroughly evaluated, and safety conclusions should be regarded with caution. Subgroup analyses revealed reduced efficacy in patients with concomitant anxiety and fibromyalgia; nonetheless, these preliminary findings necessitate validation in subsequent research.

This study offers significant preliminary evidence that LesionRF may yield superior and more sustained results compared to PRF in the management of chronic migraines. Multicenter randomized controlled studies with prolonged follow-up are necessary to establish generalizability and verify safety.

## Figures and Tables

**Table 1:** Baseline demographic and clinical characteristics of patients. Legend: No significant differences were observed. Abbreviations: SD = standard deviation; BMI = body mass index; PRF = pulsed radiofrequency; LesionRF = thermal lesion radiofrequency.

Characteristic	PRF (n = 107)	LesionRF (n = 104)	*p* Value
Age, years (mean ± SD)	40.6 ± 11.3	39.5 ± 11.0	0.48
Female, n (%)	75 (70.1)	73 (70.2)	0.91
Migraine duration, years	≈10	≈10	0.77
BMI, kg/m^2^	25–26	25–26	0.65
Presence of comorbidity, n (%)	Balanced distribution	Balanced distribution	>0.05

**Table 2:** Primary outcomes—MMD and responder rates. Legend: LesionRF demonstrated clear superiority over PRF at 9 and 12 months. Abbreviations: MMD = monthly migraine days; CI = confidence interval; SD = standard deviation; PRF = pulsed radiofrequency; LesionRF = thermal lesion radiofrequency.

Outcome	PRF (n = 107)	LesionRF (n = 104)	Group Difference/*p* Value
MMD, 12 months (mean ± SD)	10.7 ± 3.1 days	5.9 ± 2.8 days	−4.8 days (95% CI −5.9 to −3.7), *p* < 0.001
Responder rate, 3 months (%)	74 (65–82)	88 (81–94)	*p* = 0.02
Responder rate, 12 months (%)	65 (56–74)	83 (75–89)	*p* = 0.01

**Table 3:** Secondary outcomes—pain intensity, functional, and quality-of-life scores (12 months). Legend: LesionRF achieved more pronounced improvements across all scales compared with PRF. Abbreviations: VAS = Visual Analog Scale; HIT-6 = Headache Impact Test-6; MIDAS = Migraine Disability Assessment; SF-36 = Short Form-36 Health Survey; EQ-5D = EuroQol-5 Dimensions; SD = standard deviation; PRF = pulsed radiofrequency; LesionRF = thermal lesion radiofrequency.

Measure	PRF (n = 92)	LesionRF (n = 94)	*p* Value
VAS, mean ± SD	4.2 ± 1.1	2.6 ± 0.9	<0.001
HIT-6, change (points)	−8.1	−12.3	0.002
MIDAS, change (points)	−9.4	−14.1	0.004
SF-36, change (points)	+12.2	+18.3	0.001
EQ-5D, change (points)	+10.4	+15.2	0.003

**Table 4:** Attack characteristics (12 months). Legend: LesionRF achieved greater reductions in both duration and frequency compared with PRF. Abbreviations: PRF = pulsed radiofrequency; LesionRF = thermal lesion radiofrequency; SD = standard deviation.

Characteristic	PRF (n = 92)	LesionRF (n = 94)	*p* Value
Mean attack duration, hours	−5.7 ± 2.5	−8.6 ± 4.4	<0.001
Weekly attack frequency, change	−0.93 ± 0.72	−1.62 ± 0.96	<0.001

**Table 5:** Medication use rates (baseline–12 months). Legend: The table shows changes in medication use. Reductions were observed in both groups, with more pronounced absolute decreases in the LesionRF group. Abbreviations: PRF = pulsed radiofrequency; LesionRF = thermal lesion radiofrequency.

Medication Type	PRF (%)	LesionRF (%)	*p* Value
Preventive medication	59.8 → 34.6 (−25.2)	54.8 → 27.9 (−26.9)	0.29
Acute medication	60.7 → 41.1 (−19.6)	64.4 → 39.4 (−25.0)	0.80

**Table 6:** Retreatment analysis. Legend: The risk of retreatment was significantly higher in the PRF group compared with the LesionRF group. Abbreviations: HR = hazard ratio; CI = confidence interval; PRF = pulsed radiofrequency; LesionRF = thermal lesion radiofrequency.

Outcome	PRF	LesionRF
Retreatment rate (%)	19 (95% CI 12–27)	8 (95% CI 3–14)
HR (95% CI)	2.15 (1.05–4.41)	Reference

**Table 7:** Safety profile. Legend: The table shows safety outcomes. Hematomas were observed only in the LesionRF group, but all resolved completely with conservative treatment. Abbreviations: PRF = pulsed radiofrequency; LesionRF = thermal lesion radiofrequency.

Complication Type	PRF (n = 107)	LesionRF (n = 104)	Description
Minor complications	≈5% (n ≈ 5)	≈5% (n ≈ 5)	Injection-site pain, transient dizziness
Major complications	0	2 (1.9%)	Hematoma, resolved with conservative treatment
Neurological events	0	0	None observed

**Table 8:** Subgroup analyses (responder rate, 12 months). Legend: LesionRF was superior across all subgroups, although efficacy was relatively lower in patients with anxiety and fibromyalgia. Abbreviations: PRF = pulsed radiofrequency; LesionRF = thermal lesion radiofrequency.

Subgroup	PRF (%)	LesionRF (%)	*p* Value
Male	70	84	0.19
Female	62	80	0.42
Anxiety comorbidity	60	75	0.04
Fibromyalgia	55	70	0.05

## Data Availability

The original contributions presented in this study are included in the article and Supplementary Material. Further inquiries can be directed to the corresponding author.

## Supplementary Materials

The following supporting information can be downloaded at: https://www.mdpi.com/article/10.3390/medicina61111893/s1, Table S1. Complete-case vs. Imputed Results.

## Abbreviations

BMI	Body Mass Index
CI	Confidence Interval
EQ-5D	EuroQol-5 Dimensions
GON	Greater Occipital Nerve
HR	Hazard Ratio
HIT-6	Headache Impact Test-6
ICHD-3	International Classification of Headache Disorders, 3rd edition
ITT	Intention-to-Treat
LMM	Linear Mixed Model
MCID	Minimal Clinically Important Difference
MICE	Multiple Imputation by Chained Equations
MIDAS	Migraine Disability Assessment
MMD	Monthly Migraine Days
OR	Odds Ratio
PASS	Patient Acceptable Symptom State
PRF	Pulsed Radiofrequency
QoL	Quality of Life
RF	Radiofrequency
RMDQ	Roland–Morris Disability Questionnaire
RR	Risk Ratio
SF-36	Short Form-36 Health Survey
SPG	Sphenopalatine Ganglion
VAS	Visual Analog Scale
