# Supplementary material for: Comparative Long-Term Outcomes of Pulsed and Lesion Radiofrequency of the Greater Occipital Nerve in Chronic Migraine: A 12-Month Cohort Study
Source: Medicina (Kaunas). 2025 Oct 22;61(11):1893. doi: 10.3390/medicina61111893 (PMC12654755; doi:10.3390/medicina61111893)
Supplement: Supplementary file 1 [file medicina-61-01893-s001.zip › medicina-3891902-supplementary.pdf]

Supplementary

Table S1. Complete-case vs Imputed Results.

Outcome	PRF (Complete-case) Mean ± SD	PRF (Imputed) Mean ± SD	LesionRF (Complete-case) Mean ± SD	LesionRF (Imputed) Mean ± SD	p-value (Complete-case)	p-value (Imputed)
MMD (12 months)	10.7 ± 3.1	10.6 ± 3.2	5.9 ± 2.8	6.0 ± 2.9	<0.001	<0.001
VAS (12 months)	4.2 ± 1.1	4.1 ± 1.2	2.6 ± 0.9	2.7 ± 1.0	<0.001	<0.001
HIT-6 (12 months)	-8.1 ± 2.4	-8.0 ± 2.5	-12.3 ± 3.1	-12.2 ± 3.2	0.002	0.002
MIDAS (12 months)	-9.4 ± 3.0	-9.2 ± 3.2	-14.1 ± 3.4	-13.9 ± 3.5	0.004	0.005

Legend: Supplementary Table S1 presents the comparison between complete-case and imputed analyses for major outcomes at 12 months. Results are reported as mean ± SD. No clinically meaningful differences were observed between imputed and complete-case analyses.

Abbreviations: MMD = Monthly Migraine Days; VAS = Visual Analog Scale; HIT-6 = Headache Impact Test-6; MIDAS = Migraine Disability Assessment; PRF = Pulsed Radiofrequency; LesionRF = Thermal Lesion Radiofrequency; SD = Standard Deviation.
